# Bio-Based Porous Aerogel with Bionic Structure and Hydrophobic Polymer Coating for Efficient Absorption of Oil/Organic Liquids

**DOI:** 10.3390/polym14214579

**Published:** 2022-10-28

**Authors:** Yi Huang, Yucheng Wu, Hao Tao, Bihe Yuan

**Affiliations:** 1 School of Safety Science and Emergency Management, Wuhan University of Technology, Wuhan 430070, China; 2 School of Mechanical and Electronic Engineering, Wuhan University of Technology, Wuhan 430070, China

**Keywords:** porous materials, oil absorption, hydrophobic modification, agricultural residue

## Abstract

Increasing contamination risk from oil/organic liquid leakage creates strong demand for the development of absorbents with excellent hydrophobicity and absorption capacity. Herein, bagasse was carbonized to form porous char with a special structure of array-style and vertically perforated channels, and then the activation process enlarged the pore volume of the char. With the cooperation of low-surface-energy polydimethylsiloxane and diatomaceous earth particles, the modified activated carbon aerogel (MACA) was fabricated by modifying the surface coating and mastoid structure on the bagasse char. Moreover, the MACA demonstrates high porosity oil-water separation, hydrophobicity, and considerable absorption capacity (4.06–12.31 *g*/*g*) for gasoline and various organic solvents. This work converts agricultural waste into an efficient porous adsorbent, offering a scalable and commercially feasible solution to solving the leakages of oil/organic solvents.

## 1. Introduction

With the development of industrial production, the utilization of hazardous chemicals and petroleum is increasing. Unfortunately, severe oil spills frequently occur, leading to incalculable economic losses and catastrophic consequences for the environment and human health [1]. Therefore, the great public concern for environmental risk and sustainable development has greatly necessitated the exploitation of effective disposal methods for oil spills [2].

A variety of oil/organic leakage treatment methods are ceaselessly being explored by researchers all over the world. The adsorption method has become one of the most important countermeasures for oil spill cleanup, owing to its facile operation process and decrease in pollution [3]. The traditional absorbents for oil removal involve inorganic materials, natural organic and synthetic polymer adsorbents [3,4,5,6,7,8,9,10,11]. Inorganic adsorbents such as zeolites and perlite are widely investigated because of their low cost and easy availability. However, they also possess the defect of low buoyancy [7,8]. Natural organic adsorbents such as straw and corncob are available and environmentally friendly, but the poor hydrophobicity causes these materials to absorb a large amount of water while absorbing oil [9,10]. Synthetic polymers such as polyethylene and polypropylene have excellent adsorption capacity for spilled oil because of their great hydrophobicity and lipophilicity, while their marked disadvantages are non-biodegradability and inflammability [11]. Therefore, the development of organic–inorganic composite adsorbents combined with the advantages of organic and inorganic materials should be of great significance in addressing leakages of oil/organic solvents.

Bio-materials with porous properties attract widespread attention in the field of oil/organic solvent absorption. Bagasse, an inherently porous material and type of agricultural waste, presents a naturally and vertically connected plant catheter and sieve tube that transports water and nutrients. Many studies grounded sugarcane into powder and drew the sugarcane fibers to fabricate adsorbent, ignoring its special geometrical construction and pore structure [12]. It is favorable to attempt to maintain the porous skeleton with its special vertical channel characteristics, and to convert bio-based bagasse into porous aerogel for oil/organic solvent absorption [13]. Being the lightest solid, porous aerogel with a three-dimensional (3D) network structure can be applied to absorption and thermal insulation fields [6,14,15]. In addition, the carbonization-activation of bagasse not only obtains the structure of vascular bundles, but also greatly enlarges specific surface area and porosity [16]. However, some oxygenated functional groups generated during activation cause a decrease in hydrophobicity. These functional groups can be easily functionalized and have the potential to react with many polymers [16,17].

Tremendous efforts have been made to improve surface hydrophobicity through the application of some low-surface-energy polymers [18,19,20]. In spite of the important progress in developing materials with excellent hydrophobicity for oil-water separation, there are certain drawbacks, such as applying fluoro-containing chemicals and low durability in harsh conditions, which have restricted the widespread application of these functional adsorbents [19]. With fluorine-free coatings becoming increasingly prevalent, hydrophobic polymers like polydimethylsiloxane (PDMS) have attracted a great deal of attention [21]. PDMS is one of the most widely used silicon-based hydrophobic polymers, and it also possesses biocompatible and flexible characteristics. To add, the curing technology of PDMS is mature and simple [17]. Additionally, inspired by a variety of biological surfaces such as lotus leaves and rose petals, hydrophobic surface morphology with increased surface roughness displays higher hydrophobicity through the collaboration between low-surface-energy polymer and solid particles [22,23]. Therefore, hydrophobic PDMS and diatomaceous earth (DE) particles are coated on the porous material surface, which forms a more hydrophobic mastoid structure. The high hydrophobicity of porous material contributes to a strong repellency to water while absorbing oil.

Herein, we fabricated an environmentally friendly organic–inorganic composite material. The porous material obtains high porosity and specific surface area through the carbonization-activation method. Additionally, the dual-modification of surface coating and morphology of porous material is expected to perform superior hydrophobicity. Therefore, the prepared aerogels have promising applications in the field of oil/organic solvent absorption.

## 2. Materials and Methods

### 2.1. Materials

Bagasse was acquired from a farmland in Guangxi province. Sudan IV and petroleum ether were purchased from Shanghai Aladdin Biochemical Technology Co., Ltd. (Shanghai, China). PDMS prepolymer (Sylgard 184A) and curing agent (Sylgard 184B) were produced by Dow Corning Corp. (Midland, MI, USA). Potassium hydroxide (*KOH*), xylene, carbon tetrachloride, dichloromethane, ethanol, isopropyl alcohol, methanol, hexane and sodium chloride (NaCl) were offered by Sinopharm Chemical Reagent Co., Ltd. (Shanghai, China). Gasoline was provided by China National Petroleum Corp. (Beijing, China). DE was purchased from Shijiazhuang Huabang Mineral Products Co., Ltd. (Shijiazhuang, China).

### 2.2. Preparation Procedure of Carbon Aerogels

Firstly, the sugarcane was cut into chunks with a diameter of approximately 2.5 cm and a height of 1.2 cm. The chunks were then immersed in 80 °C deionized water for 72 h to eliminate impurities and sugar, and the deionized water was renewed every 8 h. Subsequently, these chunks were dried in a vacuum freeze-dryer for 48 h to retain porous bagasse (PB). The PB monoliths were heated at a heating rate of 5 °C/min to 600 °C and held for 1 h under a nitrogen (N_2_) atmosphere. The obtained bagasse carbon aerogels (BCA) were taken out of the tube furnace after cooling to room temperature.

### 2.3. Modification of Carbon Aerogels

The BCAs were immersed in 15 wt% *KOH* aqueous solution for 24 h and were then dried in an 85 °C drying oven for 24 h. Afterward, the samples were heated to 700 °C (with a heating rate of 10 °C/min) and maintained at target temperature for 1 h under a N_2_ atmosphere. The black monoliths were then washed repeatedly with deionized water until the pH value of leachate reached 7, and they were dried at 100 °C for 12 h to attain the activated carbon aerogel (ACA). Next, the ACAs were immersed in the PDMS prepolymer-hexane solution (the ratio of PDMS prepolymer to curing agent was 10:1, containing 1 g PDMS and 1.5 g DE) for 12 h, followed by curing at 85 °C for 12 h. The immersion-curing process was repeated to obtain polymer/DE-modified activated porous material with different coating layers. The samples with 1, 2 and 3 coating layers were specified as MACA, MACA-2, and MACA-3, respectively.

### 2.4. Characterization

A JSM-IT300 scanning electron microscope (SEM, JEOL Ltd., Akishima-shi, Japan) was used to examine the morphologies and microstructures of BC, ABC, and MACA.

The water contact angle (WCA) values of samples were measured using a JY-PHb contact angle meter (Shanghai Bangyi Precision Measuring Instrument Co., Ltd., Shanghai, China) at room temperature, and at least three points were detected for each sample.

The intelligent Fourier transform infrared (FT−IR) spectrum was used to examine the surface chemical functional groups of samples on a Nicolet 6700 infrared spectrometer (Thermo Fisher Scientific Inc., Waltham, MA, USA) in a wavenumber range of 4000–500 cm^−1^.

The graphitization degree of monolithic materials was obtained on a Raman microscope spectrometer (Renishaw PLC., Wotton-under-Edge, UK) with a laser line of 514.5 nm.

The crystalline structure of samples was studied using X-ray diffraction (XRD, Bruker, Karlsure, Germany) analysis on a D8 Advance X-ray diffractometer in the range of 5° to 80°.

Thermogravimetric analysis (TGA) and derivative thermogravimetry (DTG) curves of samples were provided by using a STA 6000 simultaneous thermal analyzer (PerkinElmer Inc., Waltham, MA, USA) from 30 to 750 °C at a heating rate of 20 °C/min under air atmosphere.

The specific surface area, pore volume and pore size distribution of BCA and ACA were tested using an accelerated surface area and porosimetry system (Micromeritics Instrument Corp., Norcross, GA, USA) at 77 K.

To explore thermal insulation performance, the samples were placed on a J-104 hot disk and heated at 130 °C for 5 min, and the 5-min infrared thermal infrared video was captured by using an infrared camera (FLIR A3xxsc and A6xxsc series provided by FLIR Systems, Teledyne FLIR LLC., Wilsonville, OR, USA).

To evaluate the washing stability of the coating, washing tests were performed by immersing the MACA in different 200 mL aqueous solutions including simulated seawater (3.5 wt% NaCl), hot water (80 °C), and 0.2 wt% aqueous detergent (Chaoneng detergent, provided by Nice Group Co., Ltd. Lishui, China) under stirring for 20 cycles (40 min stirring for each cycle). After each cycle, the sample was washed and dried, and then the WCA was detected.

### 2.5. Oil and Organic Solvent Absorption Experiments

The oil/water separation test was performed using gasoline (dyed by Sudan IV), which mixed with deionized water in a beaker. The absorbents were put into the beaker and the absorption process was recorded using a digital camera.

The absorption capacities of gasoline and different organic solvents were assessed by weighting the samples before and after being absorbed with oil/organic solvents. The absorbents were immersed in various liquids (xylene, carbon tetrachloride, dichloromethane, ethanol, petroleum ether, isopropyl alcohol, methanol, hexane, and gasoline) for 5 min. The absorption capacity (*q*) of samples was defined by the following equation:

*q* = (*m_t_* − *m*_0_)/*m*_0_, (1)

where *m*_0_ and *m_t_* represent the samples’ mass before and after absorption tests, respectively.

## 3. Results and Discussion

### 3.1. Preparation Schematic of Polymer/DE-Modified Porous Material

**Figure 1:**
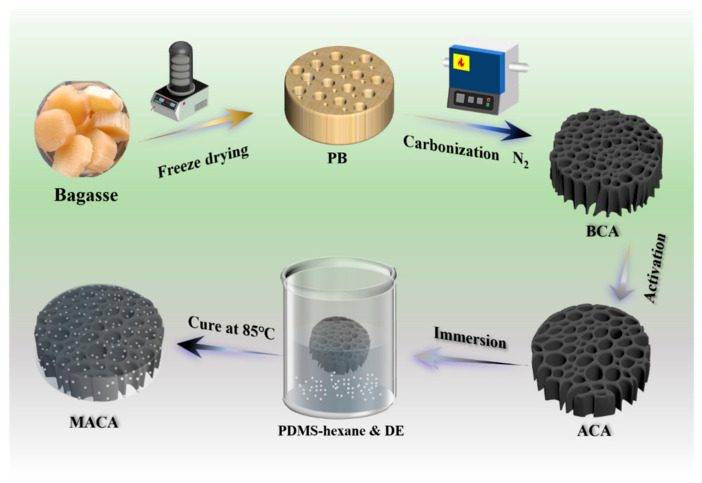
Schematic of MACA preparation.

**Figure 2:**
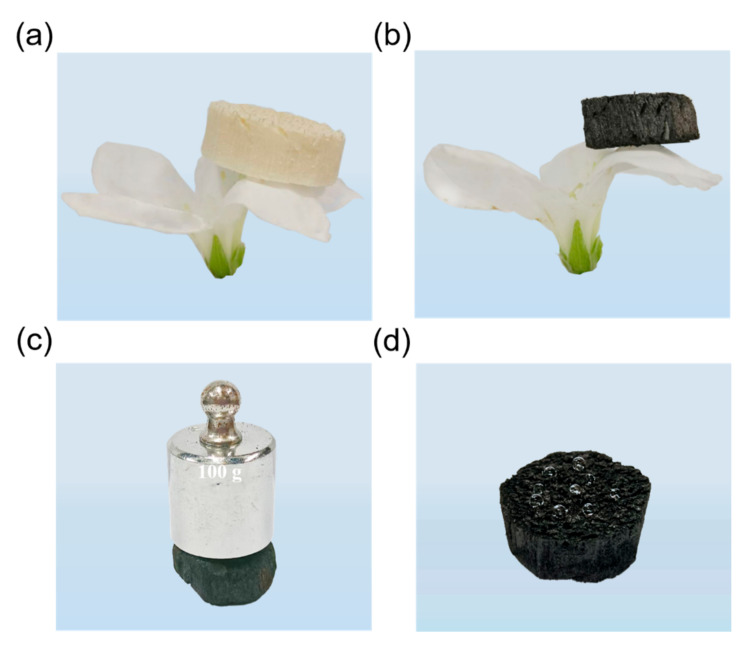
Digital images of (**a**) PB and (**b**) MACA standing on a tender petal; (**c**) A 100 g weight placed on MACA without any damage; (**d**) Quasi-spherical water droplets on the surface of MACA.

The preparation schematic of polymer/DE-modified porous material is elucidated in Figure 1. Soaking to eliminate impurity and sugar, the bagasse was subsequently treated with freeze-drying for removing its stored water to form an ultralight skeleton, which can perch on a flower without breaking its petal (Figure 2a). The robust vertical plant catheter and sieve tube structure in the xylem and phloem of sugarcane play an important role in the form-supporting and water-transporting of vascular plants. Benefitting from freeze-drying, these rigid channels used for mass transportation can be dredged. Therefore, the lightweight skeleton possesses many vertical orifices, resulting in low mass transfer resistance and capillary effect [24]. Owing to the special pore structure, the bagasse is supposed to be an oil/organic solvent absorption candidate. However, the hydrophilic property of the crudely natural absorbent hinders its utilization in the disposal of oil spills [25]. It is worth noting that the surface wettability of raw bagasse can be converted from hydrophilic to hydrophobic by removing hydrophilic groups through carbonization, and the thinner vascular bundle and larger pores can be observed [13]. Additionally, the use of *KOH* as activating agent increases the surface area and total pore volume of bagasse char. The possible reactions that occur during the activation process with *KOH* are presented as follows [16,26,27,28]:

*2KOH* → *K*_2_*O + H*_2_*O*, (2)

*C + H*_2_*O* → *H*_2_
*+ CO*, (3)

*CO + H*_2_*O* → *H*_2_
*+ CO*_2_, (4)

*K*_2_*O + CO*_2_ → *K*_2_*CO*_3_, (5)

Via strong activation with a high amount of *KOH*, carbon atoms are eliminated from the internal structure of char, which contributes to generating a more complex pore network [16,26,28]. In addition, the formation of *H*_2_*O* and *CO*_2_ during the activation process positively contributes to the further development of porosity through the gasification of carbon [28]. Through accurate coordination of the low-surface-energy polymer PDMS and DE, a lotus-leaf-like polydimethylsiloxane coating is formed on the surface of the samples, leading to excellent water repellency for the material. Thus, the polymer/DE-modified porous material is assumed to have excellent oil/solvent absorption capabilities.

### 3.2. Micro-Morphology and Structure Analyses

The prepared MACA possesses ultralight properties (with a density of approximately 0.06 g/cm^3^) and can stand on a petal (Figure 2b). In addition, the MACA can withstand at least 700-times pressure of its own weight, indicating its anti-deformation ability (Figure 2c).

**Figure 3:**
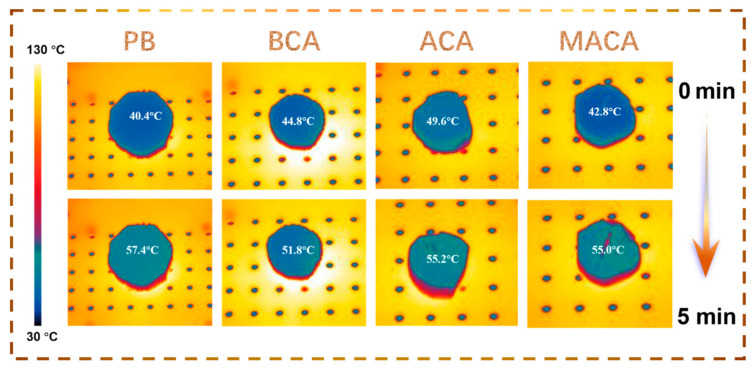
Infrared images of PB, BCA, ACA, and MACA to detect the temperature of the top surfaces of materials. The samples were put on a 130 °C hot disk for 5 min.

To evaluate thermal insulation performance, the samples were heated on a thermostatic hot plate (130 °C for 5 min). The significant temperature discrepancy between the top surface of material (<58 °C) and hot plate surface (130 °C) demonstrates the favorable thermal insulation performance of the aerogels (Figure 3). However, when comparing our results to those in previous studies, the thermal insulation performance of porous materials with special vertical holes is slightly inferior [29,30]. The main reason is that in this experiment, the aerogel was placed upright on the hot plate, and the heat was transferred vertically along the channel of this porous material from the heat source at the bottom, which easily forms thermal convection, affecting heat insulation performance.

**Figure 4:**
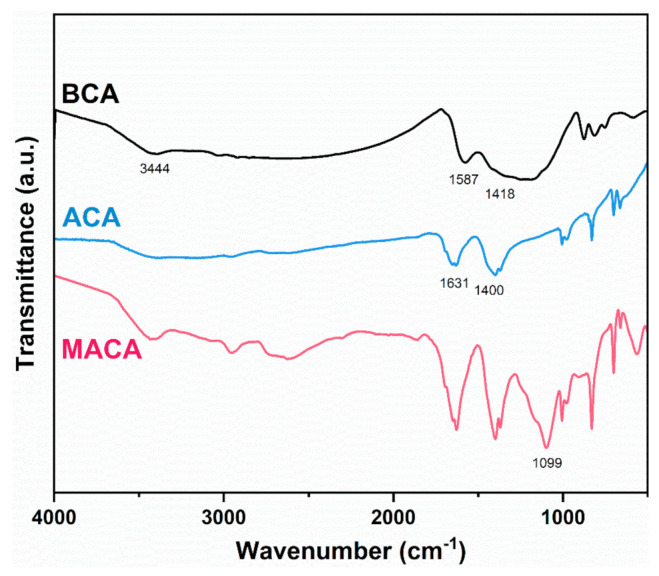
FT−IR spectra of BCA, ACA and MACA.

Figure 4 shows the FT−IR spectra of BCA, ACA, and MACA. The bagasse skeleton contains an amount of cellulose and hemicellulose, which are rich in oxygen-functional groups such as O−H at 3444 cm^−1^ [31]. The peak related to the stretching vibration of O−H is barely observed in the spectrum of BCA, indicating the occurrence of dehydration and carbonization reactions [31,32,33,34,35,36,37,38]. The absorption bands at 1587 cm^−1^ and 1418 cm^−1^ correspond to the C=C vibrations of the aromatic ring, resulting from the dehydrogenation and aromatization of saccharide molecules [31,39]. The oxygenated functional groups on ACA are produced in the *KOH* activation process [40] Thus, the adsorption peak at 1631 cm^−1^ in the spectrum of ACA is assigned to the water adhered to the material’s surface, attributing to an increase in hydrophilicity of the activated porous material [41]. Additionally, the band at 1400 cm^−1^ corresponds to the vibrations of the aromatic ring [13]. In the spectrum of MACA, a new absorption peak at 1099 cm^−1^ is attributed to the stretching vibration of Si−O−Si, which is direct evidence of the coating of PDMS [42].

**Figure 5:**
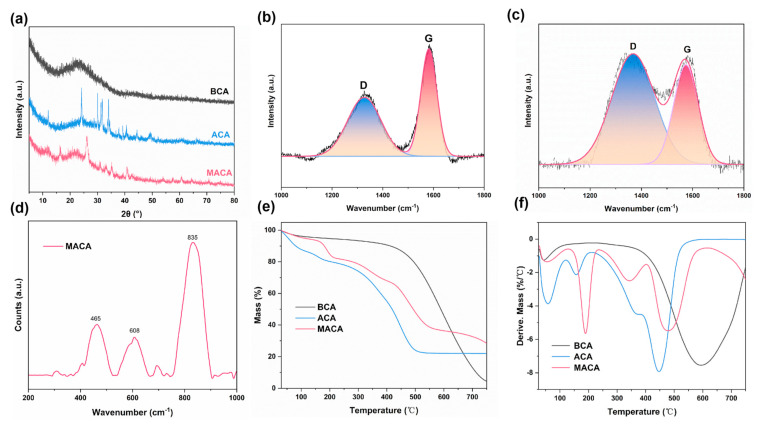
(**a**) XRD patterns of BCA, ACA, and MACA; Raman spectra of (**b**) BCA, (**c**) ACA, and (**d**) MACA; (**e**) TGA and (**f**) DTG curves of BCA, ACA, and MACA.

The crystalline structure of the samples was studied using XRD. In Figure 5a, the XRD patterns of BCA, ACA, and MACA present a similar diffraction peak at around 23°, which corresponds to the (002) crystal plane of graphitized material [43,44]. The XRD pattern of ACA shows that the (002) peak becomes weaker and broader, verifying the decreased graphitization degree after *KOH* activation [44]. Additionally, in the case of ACA and MACA, the patterns exhibit certain sharp peaks at 33° and 29° assigned to *KOH* while the peak at approximately 24° is attributed to the generated *K*_2_*CO*_3_ during *KOH* activation [45,46]. In the MACA pattern, the distinctive diffraction peak located at around 12° proves the successful adherence of PDMS [47]. Raman spectra of BCA and ACA are presented in Figure 5b,c. The graphitized carbon and amorphous carbon have two characteristic peaks located at approximately 1327 cm^−1^ (D band is attributed to disordered carbon or defective graphitic structures) and 1582 cm^−1^ (G band is related to the vibration of the sp^2^-bond carbon atoms in the graphitic layers) [44,48,49], respectively. The integral intensity ratio of G band to D band (I_G_/I_D_) of BCA (0.558) is higher than ACA (0.517), indicating the decrease in graphitization degree after *KOH* activation [13,50,51]. This result correlates previous findings in the literature and is coordinated with the analysis of XRD patterns [44]. The Raman spectrum of porous MACA is displayed in Figure 5d. The peaks at 465 cm^−1^ and 608 cm^−1^ are assigned to Si−O−Si and Si−CH_3_, respectively [52]. Additionally, CH_3_ bending appears at 835 cm^−1^, which concurs with the previously reported work on PDMS [53,54].

The thermal stability of different samples was assessed using TGA and DTG [55,56]. As plotted in Figure 5e, the initial mass loss temperature for BCA, ACA, and MACA was below 200 °C. Compared to BCA and MACA in this stage, the TGA curve of ACA exhibits the maximal mass loss, which can be attributed to the evaporation of adsorbed water and the elimination of oxygen-containing functional groups [57]. The polymer/DE-coated samples exhibit mass loss regions at 300–400 °C, which is related to the decomposition of the PDMS/DE layer [21]. The TGA curve of BCA performs the mass loss at a temperature range of approximately 400–750 °C, attributing to the oxidation reaction of carbon [58,59]. Moreover, the ACA shows the fastest mass loss rate at this phase, which can be assigned to the catalytic combustion of carbon materials by potassium salts [58,59,60]. The presence of potassium metals results in the deteriorated thermal stability of ACA [60].

**Figure 6:**
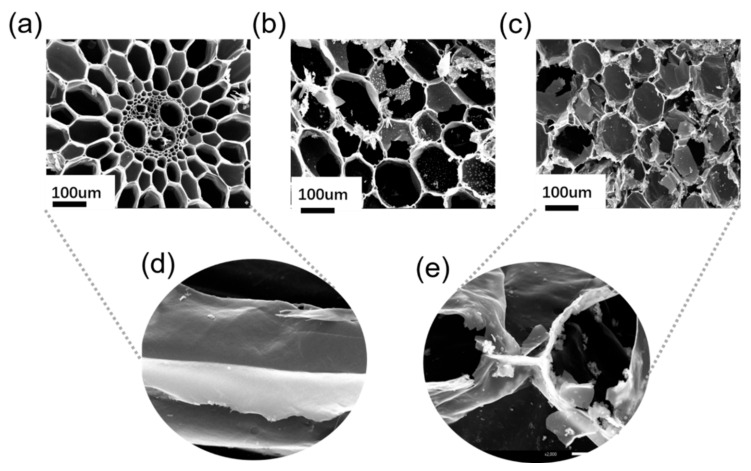
SEM images: (**a**) BCA, (**b**) ACA, (**c**) MACA, (**d**) smooth BCA inner surface, and (**e**) MACA with mastoid structure.

Figure 6 shows the SEM images of the BCA, ACA, and MACA. Notably, all samples represent porous structures with vertical channels, inheriting the hole characteristics of a vascular plant. In addition, the pore size of the BCA asymmetrically ranges from nearly 10 to 130 μm (Figure 6a). Additionally, the inner surface of the BCA is smooth (Figure 6d). According to the SEM image, the pore size of the ACA increases and has a uniform aperture distribution with an average diameter of approximately 110 μm, indicating the growth of ostioles in the *KOH* activation process (Figure 6b). With further detailed observation, there is little difference in the pore sizes between the ACA and MACA (Figure 6c). Moreover, compared to the smooth morphology of BCA, the inner surface of porous MACA is adhered some protruding DE particles, which improves the surface roughness of MACA (Figure 6e). The morphology of MACA is similar to lotus leaves (Figure S1).

**Figure 7:**
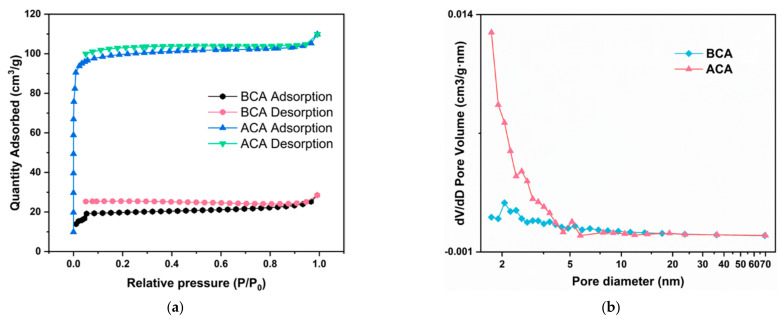
(**a**) N_2_ adsorption−desorption isotherms of BCA and ACA; (**b**) Pore size distributions (0–70 nm) of samples by using BJH model.

The N_2_ adsorption−desorption isotherm was employed to calculate the Brunauer–Emmett–Teller (BET) surface area and Barrett–Joyner–Halenda (BJH) pore size distribution of different samples (Table 1 and Figure 7) [16,61]. The specific surface area, micropore pore volume and mesopore volume of BCA are 60.99 m^2^/g, 0.03 cm^3^/g and 0.01 cm^3^/g, respectively. *KOH* activation contributes to the improvement of porosity of material [26,62]. Thus, the specific surface area, micropore pore volume and mesopore volume of ACA increase to 304.37 m^2^/g, 0.14 cm^3^/g, and 0.02 cm^3^/g, respectively. Such high specific surface area and porosity are conducive to permitting oil diffusion and transportation, enhancing the absorption capacity of porous materials. Based on the BJH method, the average pore diameter decreases from 8.98 nm (BCA) to 4.78 nm (ACA). The N_2_ adsorption−desorption isotherm of samples can be classified as a type I isotherm (Figure 7a). The isotherm linearly increases at a low relative pressure (<0.1 Pa), proving N_2_ uptake is markedly boosted in this region [63]. Therefore, these porous materials contain wide micropores and narrow mesopores, which explains the decrease in average pore diameter despite the mesoporous volume and specific surface area increase [64]. When nearly reaching the saturation pressure (>0.99 Pa), the condensation and aggregation of N_2_ in the material channel mainly cause the rise of the curves [63,65]. Additionally, in the pore size distribution curves, the ACA’s pore volumes of diameters ranging from 0 to 4 nm are higher than those of BCA (Figure 7b), further indicating the abundant coexistence of micropores and mesopores.

### 3.3. Characterization of Hydrophobicity

**Figure 8:**
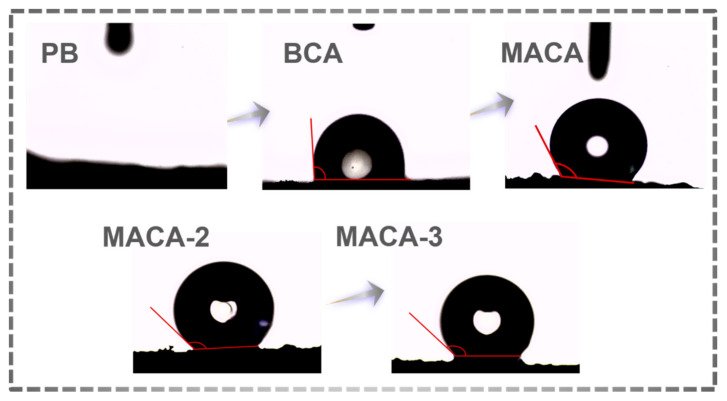
The WCA images of PB, BCA, MACA, MACA-2, and MACA-3.

**Figure 9:**
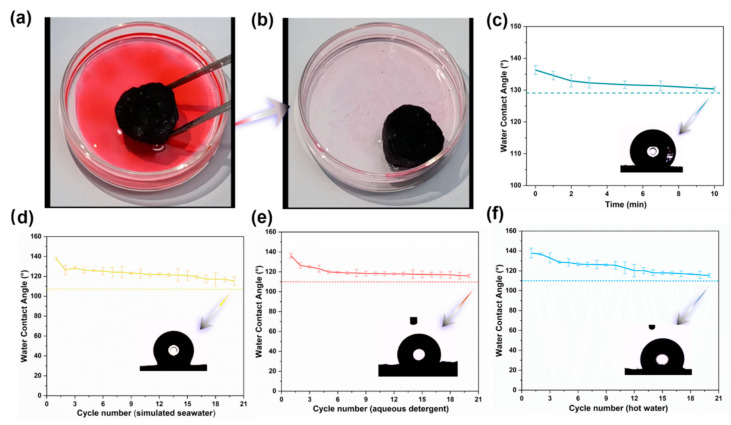
(**a**) Oil absorption test of MACA on a mixture of gasoline (dyed by Sudan IV) and deionized water; (**b**) MACA floating on the water after adsorbing the oil; (**c**) WCA values of MACA versus time; WCA values of MACA after repeated washing in (**d**) simulated seawater, (**e**) hot water and (**f**) 0.2 wt% aqueous detergent.

The surface wettability of porous material is a vital factor affecting oil-water separation applications [32]. In the modification process of porous material, the wettability of the samples causes noticeable changes (Figure 8). The original bagasse can be easily wetted using both water (Figure 8) and oil (Movie S1), directly demonstrating the natural hydrophilia of PB, whereas the hydrophobicity of BCA significantly enhances (WCA = 93.8 ± 2.1°) after carbonization [66]. The *KOH* activation process produces oxygenated functional groups from which hydrophobicity of this porous material decreases [16]. The surface hydrophobicity of porous material is improved by exerting low-surface-energy coating and enhancing the roughness of the aerogel surface, forming a hydrophobic bionic-like structure with a lotus-leaf style [34]. Movie S2 shows that when MACA was immersed in water by an external force, its hydrophilic properties allowed air bubbles trapped around it to form a silver and mirror-like surface. Additionally, when it was introduced to a mixture of water and gasoline (Figure 9a,b), MACA adsorbed oil while repelling water (Movie S3). The hydrophobicity of MACA was intuitively observed, where some water droplets stayed on the aerogel surface (Figure 2d). Although it was saturated with oil, the MACA continued to float on the water surface due to the lightweight and hydrophobicity of porous material, showing its suitability for treating oil floating on the water surface. Furthermore, a contact angle measurement was used to exactly study the influence of different coating contents on hydrophobicity. The WCA values of porous materials with different coating layers (MACA, MACA-2, and MACA-3) are 137.6 ± 0.9°, 140.7 ± 2.9°, and 145.6 ± 1.4°, respectively. With the increasing of hydrophobic coating amount, hydrophobicity performance improved slightly. To better understand the durability of this highly hydrophobic characteristic, the WCA values of MACA were tested with time variation. After 10 min, the water droplets still retained near spherical shapes (The WCA value is 130.4 ± 0.7°, Figure 9c). Additionally, to evaluate the washing stability of the coating on the carbon materials, washing tests on MACA were conducted. Figure 9d–f shows the WCA values after each cycle in different mediums. It was easily observed that the polymer/DE-modified material still performed hydrophobicity (the minimum average WCA value is 115.3°). The hydrophobic coating on the external surface was partially shed during the stirring process, resulting in a slight decrease in WCA. Conclusively, the PDMS/DE-modified porous material possesses laundering durability, indicating that the hydrophobic MACA has a bright application prospect in oil-water separation.

### 3.4. Oil/Organic Solvents Absorption Performance of Aerogels

**Figure 10:**
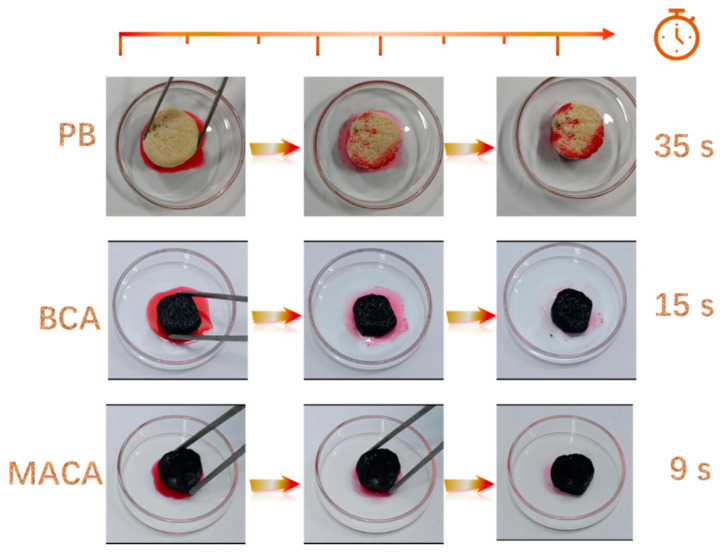
A comparison of different samples in oil absorption velocity.

**Figure 11:**
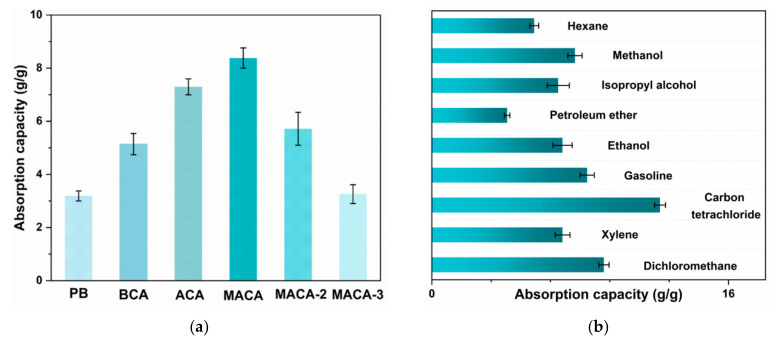
(**a**) Gasoline absorption capacities of different samples; (**b**) absorption capacities of MACA to different organic solvents and oil.

**Figure 12:**
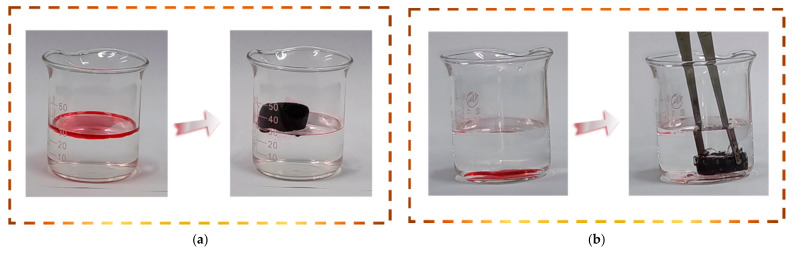
(**a**) Snapshots of the removal processes of (**a**) gasoline (dyed with Sudan IV) floating on water and (**b**) carbon tetrachloride (dyed with Sudan IV) sinking below the water by using MACA as the adsorbent.

Owing to its apparently low density, high surface hydrophobicity, high porosity, and lipophilicity, the MACA could be an ideal candidate for the absorption of oil/organic solvents. Based on this idea, absorption experiments of gasoline (dyed with Sudan IV) were conducted for demonstration purposes (Figure 10), and further detailed absorption processes are shown in Movies S1, S4 and S5. A moderate amount (approximately 2 mL) of red gasoline to each petri dish. PB, BCA, and MACA can absorb the liquid on the petri dishes, individually. Digital pictures were taken to keep track of absorption performances at different time nodes. By comparison, it was clearly noticeable that the absorption velocities of porous aerogels after hydrophobic modification were enhanced, and the entire oil could be absorbed completely by MACA within 9 s. In addition, the surface of porous MACA is similar to that of natural lotus leaves with abundant randomly distributed papillary protuberance, which improves the hydrophobicity and enhance the oil-water separation ability of porous material [32]. The adsorption capacity of polymer/DE-modified porous material greatly increased and MACA had the highest adsorption capacity of 8.38 ± 0.38 *g*/*g*, because MACA has a special bionic-like surface structure, high total pore volume, and the highest WCA (137.6° ± 0.9°). In contrast, the oil adsorption capacities of PB, BCA, and ACA are 3.19 ± 0.19, 5.14 ± 0.40, and 7.29 ± 0.30 *g*/*g*, respectively. Additionally, the coating content plays an important role in determining the absorption capacity of coated porous material. The oil absorption capacity of MACA-2 and MACA-3 decreased to 5.72 ± 0.62 and 3.26 ± 0.35 *g*/*g*, respectively (Figure 11a). The reductions can be attributed to the excess amount of polymer/DE filling the 3D-structure and pores within the material, leading to the reduced porosity of the samples (Figure S2) [21]. As the absorption process shows in Figure 12, gasoline floating on the water surface and tetrachloromethane sinking under the water were absorbed to further identify the oil-water separation capability of MACA (Movie S6–S7). This experiment indicates that MACA can be used to separate oil and water effectively without complicated operations. Furthermore, another three organic solvents were selected to test the universality of absorption. MACA exhibits high adsorption capacities (Table S1), and the absorptive amounts are 7.05 ± 0.52, 12.31 ± 0.30, 9.28 ± 0.28, 7.05 ± 0.52, 4.06 ± 0.15, 6.81 ± 0.60, 7.72 ± 0.38, and 5.52 ± 0.24 *g*/*g* for xylene, carbon tetrachloride, dichloromethane, ethanol, petroleum ether, isopropyl alcohol, methanol, and hexane, respectively (Figure 11b).

## 4. Conclusions

We successfully synthesized a new type of hydrophobic-activated carbon aerogel by using biochar derived from bagasse. This bagasse-derived porous carbon aerogel with an array-style and vertically perforated channels possesses higher porosity and a larger specific surface area and pore volume after *KOH* activation. Additionally, attributing to a powerful combination between low-surface-energy polymer PDMS and improved surface roughness on the surface of MACA, both the hydrophobicity and absorption capacities of porous aerogel are enhanced. The porous MACA can selectively absorb oil in an oil/water mixture and exhibits high adsorption capacities for gasoline (8.38 ± 0.38 *g*/*g*) and organic solvents (4.06–12.31 *g*/*g*). Overall, this novel hydrophobic absorbent with unique design concepts and high absorption efficiency exhibits a promising application at the disposal of leakages of oil/organic solvents in different complex conditions.

## Figures and Tables

**Table 1:** Pore properties of BCA and ACA.

Sample	S_BET_ ^1^	S_Micro_ ^2^	S_Meso_ ^3^	V_Total_ ^4^	V_Micro_ ^5^	V_Maso_ ^6^	D_BJH_ ^7^
BCA	60.99	52.30	8.70	0.04	0.03	0.01	8.98
ACA	304.37	274.12	30.25	0.16	0.14	0.02	4.78

^1^ BET surface area. ^2^ t-plot external surface area [micropore surface area]. ^3^ t-plot external surface area, [mesopore surface area] was calculated by total surface area (BET surface area) −t-plot micropore surface area. ^4^ Total pore volume. ^5^ t-plot micropore volume. ^6^ Mesopore volume. ^7^ BJH adsorption average pore diameter.

## Data Availability

The data presented in this study are available on request from the corresponding author.

## Supplementary Materials

The following supporting information can be downloaded at: https://www.mdpi.com/article/10.3390/polym14214579/s1, Movie S1: PB absorbed (approximately 2 mL) oil from the petri dish in 45 s; Movie S2: When MACA was immersed in water by an external force, its hydrophilic properties allowed air bubbles trapped around it to form silver and mirror-like surface; Movie S3. When it was put into a mixture of water and gasoline, MACA adsorbed oil while repelling water; Movie S4. BCA absorbed (approximately 2 mL) oil from the petri dish in 15 s; Movie S5: MACA absorbed (approximately 2 mL) oil from the petri dish in 9 s; Movie S6: MACA absorbed gasoline floating on the water’s surface; Movie S7. MACA absorbed carbon tetrachloride sinking under the water surface; Figure S1: SEM image of the lotus leaf; Figure S2: SEM image of MACA-2; Table S1: Oil and organic solvents absorption capacities of different absorbents.
