# Supplementary material for: Bio-Based Porous Aerogel with Bionic Structure and Hydrophobic Polymer Coating for Efficient Absorption of Oil/Organic Liquids
Source: Polymers (Basel). 2022 Oct 28;14(21):4579. doi: 10.3390/polym14214579 (PMC9658809; doi:10.3390/polym14214579)
Supplement: Supplementary file 1 [file polymers-14-04579-s001.zip › Supplementary Material.pdf]

Supplementary Material

Bio-based porous aerogel with bionic structure and hydrophobic polymer coating for efficient absorption of oil/organic liquids

Yi Huang,[1] Yucheng Wu,[1] Hao Tao,[2] Bihe Yuan[1,*]

[1] *School of Safety Science and Emergency Management, Wuhan University of Technology, Wuhan 430070, China*

[2] *School of Mechanical and Electronic Engineering, Wuhan University of Technology, Wuhan 430070, China*

Movies

Movie S1. PB absorbed (approximately 2 mL) oil from the petri dish in 45 s.

Movie S2. When MACA was immersed in water by an external force, its hydrophilic properties allowed air bubbles trapped around it to form silver and mirror-like surface.

Movie S3. When it was put into a mixture of water and gasoline, MACA adsorbed oil while repelling water.

Movie S4. BCA absorbed (approximately 2 mL) oil from the petri dish in 15 s.

Movie S5. MACA absorbed (approximately 2 mL) oil from the petri dish in 9 s.

Movie S6. MACA absorbed gasoline floating on the water's surface.

Movie S7. MACA absorbed carbon tetrachloride sinking under the water surface.

Figure captions

Figure S1. SEM image of the lotus leaf.

Figure S2. SEM image of MACA-2.

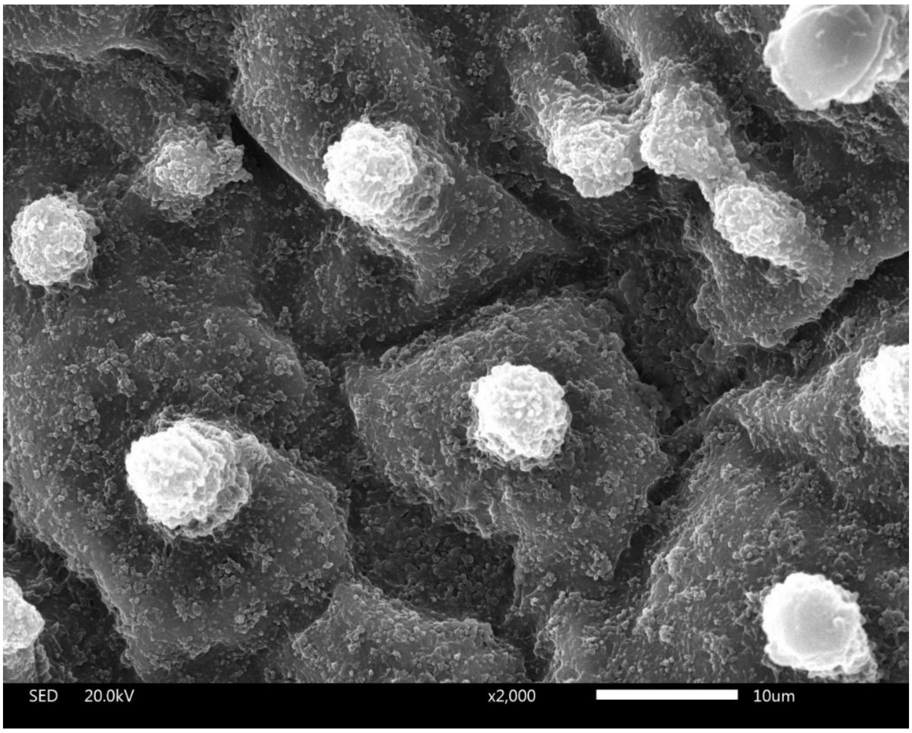

Figure S1. SEM image of the lotus leaf.

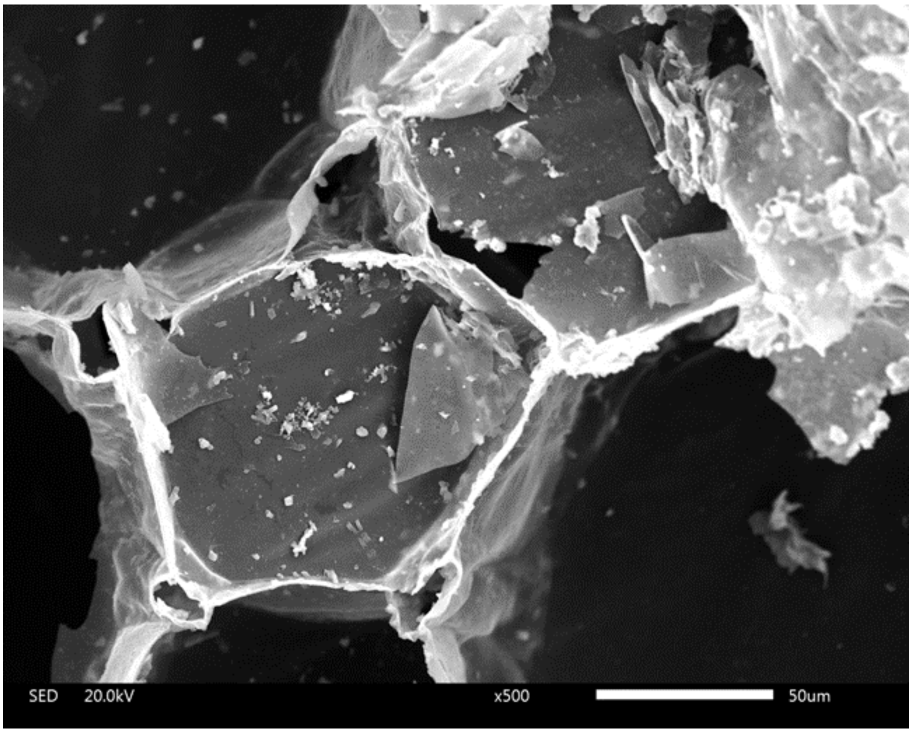

Figure S2. SEM image of MACA-2.

Table caption

Table S1. Oil and organic solvents absorption capacities of different absorbents.

Absorbent material	Collected substances	Absorption capacity (g/g)	WCA (°)	Refs
PDMS-modified carbonaceous aerogel	Crude oil, diesel, n-hexane, peanut oil and engine oil	3.3-11.4	144.2	[1]
Carbon aerogel derived from durian shell	DMF, ethanol, acetone, p-Xylene, n-heptane, toluene, methanol, formic acid, NMP, pump oil, castor oil, sunflower oil and soybean oil	3-19	112.3	[2]
Carbon spheres derived from the fruit of Liquidambar formosana	Turpentine oil, paraffin oil, methyl silicone oil, soybean oil and N-Methyl-2-pyrrolidone	2-3	/	[3]
PLA nonwoven coated with dopamine/SiO_2/polystyrene (PS)	Hexane, toluene, carbon tetrachloride, pump oil and vegetable oil	4-11	125.2 ± 1.3	[4]
Iron oxide nanoparticles sorbent	Lubricating oil	6.1-7.2	/	[5]
Polypropylene melt blown nonwoven	Motor oil and soybean oil	11.91-13.31	137	[6]
Polypropylene nonwoven with different prepared method	Soybean oil and motor oil	5.7-13.31	135	[7]
PDMS sponge	Chloroform, dichloromethane, 1,2-dichlorobezene, silicone oil, motor oil, N,N-dimethylmethane, toluene, transformer oil, methanol, ethanol and acetone	4-11	> 120-130	[8]
MACA	N-hexane, xylene, gasoline, carbon tetrachloride, dichloromethane, ethanol, isopropyl alcohol, methanol, hexane and petroleum ether	4.06-12.31	137.6±0.9	This study

References:

1. Wang, Z.; Jin, P.; Wang, M.; Wu, G.; Dong, C.; Wu, A. Biomass-Derived Porous Carbonaceous Aerogel as Sorbent for Oil-Spill Remediation. *ACS Appl Mater Interfaces* **2016**, *8*, 32862-32868.

2. Wang, Y.; Zhu, L.; Zhu, F.; You, L.; Shen, X.; Li, S. Removal of organic solvents/oils using carbon aerogels derived from waste durian shell.*J. Taiwan Inst. Chem. Eng.* **2017**, *78*, 351-358.
3. Feng, Y.; Liu, S.; Liu, G.; Yao, J. Facile and fast removal of oil through porous carbon spheres derived from the fruit of Liquidambar formosana. *Chemosphere* **2017**, *170*, 68-74.
4. Zhu, C.; Jiang, W.; Hu, J.; Sun, P.; Li, A.; Zhang, Q. Polylactic Acid Nonwoven Fabric Surface Modified with Stereocomplex Crystals for Recyclable Use in Oil/Water Separation. *ACS Appl. Polym. Mater.* **2020**, *2*, 2509-2516.
5. Kumar, A.; Sharma, G.; Naushad, M.; Thakur, S. SPION/β-cyclodextrin core–shell nanostructures for oil spill remediation and organic pollutant removal from waste water. *Chem. Eng. J.* **2015**, *280*, 175-187.
6. Alassod, A.; Abedalwafa, M.A.; Xu, G. Evaluation of polypropylene melt blown nonwoven as the interceptor for oil. *Environ. Technol.* **2021**, *42*, 2784-2796.
7. Alassod, A.; Xu, G. Comparative study of polypropylene nonwoven on structure and wetting characteristics. *J. Text. Inst.* **2020**, *112*, 1100-1107.
8. Choi, S.J.; Kwon, T.H.; Im, H.; Moon, D.I.; Baek, D.J.; Seol, M.L.; Duarte, J.P.; Choi, Y.K. A polydimethylsiloxane (PDMS) sponge for the selective absorption of oil from water. *ACS Appl. Mater. Interfaces* **2011**, *3*, 4552-4556.
